# EpCAM (CD326) finding its role in cancer

**DOI:** 10.1038/sj.bjc.6603494

**Published:** 2007-01-09

**Authors:** P A Baeuerle, O Gires

**Affiliations:** 1 Micromet, Inc., 2110 Rutherford Road, Carlsbad, CA, 92008, USA; 2 Clinical Cooperation Group Molecular Biology, GSF Research Center for Environment and Health, and Department for Head and Neck Research, Ludwig-Maximilians-University, Marchioninistr. 15, Munich 81377, Germany

**Keywords:** EpCAM, CD326, meeting report, role in cancer

## Abstract

Although epithelial cell adhesion/activating molecule (EpCAM/CD326) is one of the first tumour-associated antigens identified, it has never received the same level of attention as other target proteins for therapy of cancer. It is also striking that ever since its discovery in the late 1970s the actual contribution of EpCAM to carcinogenesis remained unexplored until very recently. With a First International Symposium on EpCAM Biology and Clinical Application this is now changing. Key topics discussed at the meeting were the frequency and level of EpCAM expression on various cancers and its prognostic potential, the role of EpCAM as an oncogenic signalling molecule for cancer cells, recent progress on EpCAM-directed immunotherapeutic approaches in clinical development and the interaction of EpCAM with other proteins, which may provide a basis for a therapeutic window and repression of its growth-promoting signalling in carcinoma. Future research on EpCAM may benefit from a unified nomenclature and more frequent exchange among those who have been working on this cancer target during the past 30 years and will do so in the future.

The two-day meeting gathered participants from academic institutions, biotech, and pharmaceutical industry, and gave an excellent opportunity and framework to review the progress on epithelial cell adhesion and activating molecule (EpCAM) biology and the development of novel EpCAM-directed immunotherapies.

In his introductory remarks, G Riethmueller (Munich, Germany) pointed out that the first human tumour-associated antigen identified with monoclonal antibodies (mAb) was in fact EpCAM or 17-1A antigen (Herlyn *et al*, 1979). Moreover, the first monoclonal antibody ever applied for human cancer therapy was murine mAb 17-1A recognising EpCAM (Sears *et al*, 1982, 1984). In 1994, mAb17-1A (later named edrecolomab and Panorex®) was also the first to show clinical efficacy in a human cancer indication in terms of prolonged overall survival (Riethmüller *et al*, 1994). It was then almost 30 years after the first generation of an EpCAM-specific monoclonal antibody that an international community gathered in Bavaria to discuss for the first time the most recent advances in the field of EpCAM research and clinical utility.

EpCAM has meanwhile advanced to an established epithelial cell marker used in pathological examinations. Likewise, EpCAM can now be considered to be one of the most frequently and most intensely expressed tumour-associated antigens known. It is found expressed on a great variety of human adenocarcinoma and squamous cell carcinoma (Went *et al*, 2004). Very recent immunohistochemistry (IHC) studies analysed fairly large sample numbers from patients with breast, prostate, ovarian, lung, colon, renal, and gastric cancer (Spizzo *et al*, 2004, 2006b; Went *et al*, 2005, 2006). These data impressively underscore the potential utility of EpCAM as immunotherapeutic target for treatment of the most frequent human cancers.

At least seven different immunotherapeutic approaches are currently in clinical trials that target EpCAM (see Table 1). Some of them have already progressed to the level of phase II and pivotal trials, indicating that initial safety questions with EpCAM-directed therapeutic approaches have been successfully addressed in preceding phase 1 studies and that the assessment of clinical efficacy is now of primary interest.

Only recently, research on EpCAM started to focus on its role in carcinogenesis. In 2004, first evidence for a direct role of EpCAM in growth-promoting nuclear signalling was reported (Munz *et al*, 2004). These data and results published by the group of Gillanders (Osta *et al*, 2004) revived research on the biological function of EpCAM, which is now delivering breakthrough results.

The present meeting review is focused on new data investigating the significance of EpCAM for cancer biology and therapy, and can therefore not adequately cover all presentations given at the symposium. We apologise to those attendees whose presentations are not mentioned or not fully covered. The majority of the meeting presentations can be viewed at www.epcam-symposium.de.

## 

### NEED FOR A COMMON NAME OF THE PROTEIN

The human EpCAM protein has plenty of synonyms, including Ep-CAM, 17-1A, HEA125, MK-1, GA733-2, EGP-2, EGP34, KSA, TROP-1, ESA, and KS1/4, among others. This is due to the fact that the protein has been independently discovered many times as a highly immunogenic tumour-associated antigen. With each discovery, the antigen received the name of the respective monoclonal antibody recognising it. Cloning of the EpCAM gene and respective analyses of antigens finally, verified, in each case, their identity with EpCAM. Nevertheless, different protein names are still being used today, which makes it difficult even for the specialist to follow the latest publications, whereas it is nearly impossible for the nonspecialist to obtain a comprehensive picture of the properties of this tumour-associated antigen and its therapeutic potential.

The attendees of the symposium therefore unanimously wish to suggest to the scientific community to use for future reference only one name for this protein. This name is proposed to be EpCAM (without a dash) as an abbreviation for the ability of the protein to act as an epithelial-specific cell adhesion molecule (Litvinov *et al*, 1994) and activating molecule (Munz *et al*, 2004; Osta *et al*, 2004). It is also suggested to add in brackets on the first occurrence of the EpCAM name in a text the recently introduced cellular differentiation (CD) marker nomenclature for EpCAM, namely CD326.

EpCAM (CD326) is related to a protein called TROP-2 showing approximately 50% sequence identity. Because TROP-2 is much less investigated than EpCAM in terms of function and tissue distribution, the attendees agreed that, for now EpCAM should not be termed EpCAM-1, and TROP-2 not EpCAM-2, until this is justified by further data supporting a similar function and tissue tropism of the two proteins.

Recently, the gene for EpCAM was renamed from GA733-2 to TACSTD1, the abbreviation for ‘tumour-associated calcium signal transducer protein 1-precursor’. It is also suggested to abandon this gene name as it does not properly reflect the function of the encoded protein and to either revert to the previous gene name, or adopt a new name, for example *epcam*.

## EPCAM STRUCTURE AND FUNCTION

**Figure 1:**
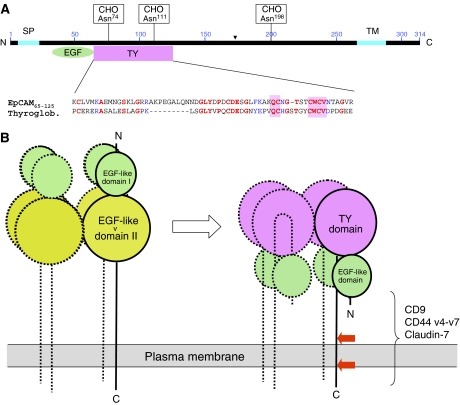
Revised structural model of EpCAM. (**A**) Sequence homology between the second EGF-like domain of EpCAM and a repeat from human thyroglobulin (TY). EpCAM is shown with N- (N) and C-terminus (C), signal peptide (SP), transmembrane domain (TM), N-linked carbohydrates (CHO), EGF-like domain (EGF) and TY repeat domain (TY). Sequence identity (red amino acid residues) between EpCAM and a selected TY repeat is 54%. Conserved exchanges are shown in blue. The cognate motif of all TY domains, QCN_x_CWCV, is highlighted by pink boxes. (**B**) Old (left) and revised domain model of EpCAM (right). EpCAM is depicted as a tetramer showing three additional subunits with dotted lines. Structural analysis of many TY domains from different proteins has shown that N- and C-termini are in close proximity. This is why the polypeptide chain is depicted with a bent within the TY domain that would orient the EGF-like domain towards the cell membrane. Cleavage sites for two proteases releasing the intracellular portion of EpCAM are indicated by red arrows, and the domain of EpCAM interacting with the listed proteins is shown by a bracket.

EpCAM is a type I membrane protein of 314 amino acids (aa) of which only 26 aa are facing the cytoplasm. The extracellular domain is believed to contain two epidermal growth factor (EGF)-like domains. P Baeuerle (Carlsbad, USA) presented results corroborating earlier reports showing that the second EGF-like repeat of EpCAM is in fact a thyroglobulin (TY) repeat domain (Linnenbach *et al*, 1989; Chong and Speicher, 2001) (Figure 1A). Thyroglobulin domains are frequently mistaken as EGF-like domains (Novinec *et al*, 2006). An accordingly revised structure model for EpCAM is shown in Figure 1B, which also includes additional features of EpCAM as reported during this symposium. A common biological function of all TY domains investigated to date is that they can potently inhibit cathepsins, a family of cysteine proteases frequently produced by tumour cells and known to be involved in metastasis (Nomura and Katunuma, 2005). Ongoing studies are investigating a role of EpCAM as membrane-bound protease inhibitor, a function that may serve to protect tumour cells from their own secreted cathepsins during metastasis.

D Maetzel (Munich, Germany) reported on the glycosylation status of EpCAM. Point mutation analysis of potential *N*-glycosylation sites verified that all three sites in EpCAM are modified in human epithelial cells (see Figure 1A) in contrast to data claiming the lack of glycosylation at Asp^198^ (Chong and Speicher, 2001). Studies with tumour samples from head and neck cancer patients indicate that EpCAM can be aberrantly glycosylated on tumour cells (Pauli *et al*, 2003). The nature and functional implications of these carbohydrate modifications awaits further analysis.

## EPCAM AS ONCOGENIC SIGNALLING PROTEIN

As reviewed by W Gillanders (St Louis, USA), EpCAM overexpression on breast cancer cell lines is mandatory for proliferation, migration, and invasiveness of tumour cells (Osta *et al*, 2004). Knockdown of EpCAM expression by specific short interfering RNA had profound effects on the expression of certain genes. Although expression of *α*-catenin mRNA was enhanced, EpCAM knockdown reduced expression of c-myc, cyclin D and survivin, supporting a proliferative signalling activity of overexpressed EpCAM.

A highlight of the symposium was new data presented by M Munz (Munich, Germany, manuscript submitted) that unravelled the entire pathway of EpCAM signalling from the cell membrane into the nucleus. Epithelial-specific cell adhesion activation molecule signalling was found induced either by soluble EpCAM or through zones of cell–cell contact, suggesting that oligomerisation is a trigger for EpCAM signalling. These signals led to cleavage of EpCAM by proteases, releasing an intracellular peptide termed EpIC. Involvement of proteases in EpCAM signalling was supported by both their co-precipitation with EpCAM and by the inhibitory effects of specific small molecule protease inhibitors, which also reduced EpCAM-mediated gene expression events.

Two-hybrid screening identified adaptor proteins as intracellular receptors for EpIC allowing the formation of a multiprotein signalling complex shown to be involved in target gene induction and growth-promoting effects. Multicolour immunofluorescence analysis impressively demonstrated cytoplasmic/nuclear redistribution of all the involved proteins depending on EpCAM signalling. An antibody to EpIC was found to give a speckled staining inside the nucleus of stimulated cells, indicating an association of the EpCAM signalosome with specific chromatin sites. With specific inhibitors of EpCAM cleavage, a pharmacological means is given to interfere with EpCAM signalling in tumour cells. Future studies are needed to understand how EpCAM signalling is regulated in normal *vs* malignant cells.

Support for a mitogenic signalling of EpCAM came from experiments in which EpCAM was overexpressed under control of the MMTV LTR in mammary glands of transgenic mice (S Litvinov, Amsterdam, The Netherlands). Glands of virgin 1.5-year-old mice had hugely overgrown ducts that were dilated, showed extensive budding, and produced milk proteins. Reduced apoptosis and a high-level Bcl-2 expression, as well as increased proliferation (Ki67 marker), were noted.

## EPCAM ON DTC

Disseminated tumour cells (DTC) can be detected in bone marrow of cancer patients using a pan-cytokeratin (CK) antibody, as reviewed by K Pantel (Hamburg, Germany). Numerous studies have shown that the occurrence and number of CK^+^ DTC in bone marrow of various cancers correlates with a poor survival prognosis of patients (Braun *et al*, 2005; Janni *et al*, 2005; Muller and Pantel, 2005; Muller *et al*, 2005; Pantel and Woelfle, 2005). A proportion of DTC in bone marrow is also positive for EpCAM and their depletion in breast cancer patients has been demonstrated by anti-EpCAM monoclonal antibody edrecolomab. Occurrence of EpCAM^+^ DTC in lymph nodes and in peripheral blood samples of cancer patients has also been shown to correlate with poor survival. A validated platform for detection and quantitation of circulating EpCAM^+^ tumour cells (CTC) has been established and is now being explored for therapy monitoring (Rao *et al*, 2005; Woelfle *et al*, 2005). The employment of enrichment beads using an anti-EpCAM antibody in addition to an anti-HER-2 antibody, did not apparently isolate CTC with an altered phenotype and expression profile.

C Klein (Munich, Germany), a pioneer in the study of single DTC for genomic aberrations and transcriptional patterns (Klein *et al*, 2002a, 2002b), investigated subpopulations of tumour cells as found in bone marrow biopsies of prostate cancer patients. In these patients staged as M0 (no metastasis), BR (biochemical PSA relapse), and M1 (established metastasis), EpCAM-positivity of *bona fide* tumour cells significantly increased with progression from M0 to BR, and further to M1 stages from 9 to 16–33%. Epithelial-specific cell adhesion activation molecule^+^ tumour cells had double the amount of chromosomal aberrations than CK^+^ tumour cells, and these aberrations affected different chromosome locations. There was only a small overlap between EpCAM^+^ and CK^+^ DTC populations of 9.5%. Epithelial-specific cell adhesion activation molecule thus defined a subpopulation of DTC in prostate cancer patients that – unlike CK^+^ DTC – already expanded during biochemical relapse and had a phenotype different from that of CK^+^ tumour cells.

Expression of EpCAM on DTC and CTC, which are suspected to include early progenitor cells for metastases, is consistent with a role of EpCAM in tumour growth and progression, and stem cell fate. Future studies need to investigate and corroborate whether EpCAM is a marker for highly tumorigenic cancer stem cells, as has recently been suggested (Al-Hajj *et al*, 2003).

## EPCAM EXPRESSION AND SURVIVAL PROGNOSIS OF CANCER PATIENTS

Another topic of the symposium was the question why EpCAM expression had in some tumour types a negative, in most a seemingly neutral and in other tumour types, a positive prognostic impact on the overall survival of patients (Table 2). P Went (Basel, Switzerland) presented data from analyses of hundreds to thousands of samples of prostate, lung, colon, gastric, and renal cell cancer patient for the frequency, intensity, and homogeneity of EpCAM expression using highly standardised staining conditions on tissue microarrays (Went *et al*, 2004, 2005, 2006). On average, high-level and mostly homogenous EpCAM expression was found on 85% of adenocarcinoma and on 72% of squamous cell carcinoma. In prostate cancer, a subanalysis indicated that EpCAM expression is higher on hormone-refractory tumour tissue than on earlier stages (*P*=0.015), and that there is a decrease in EpCAM expression with increasing Gleason score (*P*=0.011). In the subgroup of pT2 adenocarcinoma of the lung, a high EpCAM expression was correlated with a better prognosis of patients.

G Gastl (Innsbruck, Austria) reviewed the expression of EpCAM in human breast cancer and its strong negative correlation with survival parameters in node-positive patients (Spizzo *et al*, 2004). Efforts for standardising the immunohistochemical staining and evaluation procedure were explained highlighting an advanced scoring system that reflected both frequency, and intensity of staining. A similar negative correlation between EpCAM expression and survival as previously observed by the group for breast and gall bladder cancer (Varga *et al*, 2004) was also now observed with ampullary carcinoma of the pancreas (Fong *et al*, 2006) and for squamous cell cancer of head and neck.

N Stoecklein (Duesseldorf, Germany) reported a negative correlation between EpCAM expression and overall survival in patients with squamous cell carcinoma of the oesophagus, a very deadly disease with no established systemic treatment option. Although normal epithelium showed no EpCAM expression, up to 41% of patients showed high-level expression with a strong negative prognostic impact (*P*=0.0002) (Stoecklein *et al*, 2006).

A number of studies aim at the understanding of the regulation of EpCAM at the transcriptional level. These studies include deletion analysis of the EpCAM promoter and identification of transcription factors governing EpCAM expression (O Gires, Munich, W Gommans, and I van der Gun, The Netherlands) and analysis of the methylation and amplification status of the EpCAM gene (G Spizzo, Innsbruck, Austria). No differences in promoter methylation could be found in breast cancer *vs* normal tissue samples, albeit studies in breast cancer cell lines suggested a role for methylation in the regulation of EpCAM expression (Spizzo *et al*, 2006a).

M Mitas (Charleston, USA) presented data from screening for genes commonly expressed in metastatic lymph nodes from lung, breast, and pancreas cancers but not in normal lymph nodes. Epithelial-specific cell adhesion molecule and activation molecule, TROP-2, CK8, CK19, and claudin-3 were among the few commonly expressed genes, which fell into two clusters. Upregulation of Ets family transcription factor Esx/Elf3 in metastatic lymph nodes correlated well with expression of EpCAM, and is being investigated as potential transcriptional regulator.

## EPCAM-DIRECTED IMMUNOTHERAPIES UNDER DEVELOPMENT

G Schlimok (Augsburg, Germany) reviewed the clinical development of the anti-EpCAM mAb edrecolomab (17-1A; Panorex®). This well-tolerated murine IgG2a antibody, which was temporarily marketed in Germany, showed a significant increase in overall survival of colorectal cancer patients in the adjuvant setting in two out of four trials (Riethmüller *et al*, 1994, 1998; Hempel *et al*, 2000; Punt *et al*, 2002; Hartung *et al*, 2005). The speaker concluded that despite a very benign safety profile of edrecolomab, its short half-life, rapid neutralisation by a mouse-anti-human antibody response, and a borderline clinical activity asks for development of an improved mAb, ideally of human nature. H Loibner (Vienna, Austria) reviewed clinical trials using very low doses of edrecolomab as a subcutaneously administered vaccine, called IGN101, for induction of an anti-idiotypic antibody response. Virtually all patients developed humoral immune responses against the vaccine antigen, and a reduction of circulating tumour cells was observed in Phase I trial. A double-blind placebo-controlled phase II trial in several carcinoma indications did not reveal a significant impact on overall survival, but retrospective analysis of a subgroup of the stage IV rectal cancer patients showed a significant survival benefit (poster published at ASCO, 2005). A large double-blind placebo-controlled phase II/III trial in adjuvant non-small-cell lung cancer (NSCLC) is ongoing.

P Baeuerle (Carlsbad, USA) described adecatumumab (MT201), a fully human anti-EpCAM antibody (Naundorf *et al*, 2002) that currently is in three ongoing clinical trials: two phase II studies with metastatic breast cancer and early-stage prostate cancer patients, respectively, and a phase I study testing the safety of a combination with taxotere. A completed phase I study in hormone-resistant prostate cancer patients has shown that the human antibody was well tolerated at all doses tested, has not reached a maximum tolerated dose at 6 mgkg^−1^, and showed no signs of pancreatitis, which had been an issue with two high-affinity anti-EpCAM mAbs. Preclinical examination has shown that adecatumumab mainly acts through antibody-dependent cellular (ADCC) and complement-dependent cytotoxicity (CDC), and shows high antitumour activity in a lung metastasis mouse model when equipped with a murine Fc portion of the *γ*2A subtype, which is better compatible with the murine immune system than the human *γ*1 subtype. The speaker also presented preclinical data on MT110, a single-chain EpCAM/CD3-bispecific antibody construct of the BiTE class (Brischwein *et al*, 2006). In immunodeficient mice, very low doses of MT110 led to the eradication of established tumours derived from human cancer cell lines mixed with human T cells, as well as ovarian cancer tissue of patients. In the latter case, the sole source of human T cells was the xenograft itself, indicating that MT110 can penetrate human tumour xenografts after intravenous (i.v.) injection and reactivate the low numbers of tumour-resident T cells to effectively eliminate the metastatic tissue.

H Lindhofer (Munich, Germany) reviewed preclinical and clinical data of catumaxomab, a hybrid mouse IgG2a/rat IgG2b antibody that is trispecific for EpCAM, CD3 and -via its Fc portion- for antigen-presenting as well as a variety of other cytotoxic immune cells. In mouse tumour models the trioma format shows high antitumour activity and survival of which part is due to a vaccination effect (Lindhofer *et al*, 1996). When intraperitoneally (i.p.) given to ovarian cancer patients suffering from ascites and peritoneal carcinomatosis, a pronounced clinical activity was observed that in a phase I trial 21 out of 23 patients were freed of malignant ascites. A rapid clearance of essentially all EpCAM-positive tumour cells in ascites fluid and an expansion of T-cell numbers were observed, which was followed by a retraction of immune cell counts.

A pivotal study of catumaxomab in ovarian cancer patients with malignant ascites has already enroled more than 250 patients. A phase II study is ongoing with gastric cancer patients who are treated during gastric surgery for prevention of peritoneal carcinomatosis. Hence, catumaxomab appears as a very promising biological agent for the local treatment and prevention of peritoneal carcinomatosis.

A variation of the trispecific approach was presented by G Moldenhauer (Heidelberg, Germany). Hybrid antibodies containing one half of the EpCAM-specific antibody HEA125 and one half of the anti-CD3 antibody OKT3 were generated by the hybrid-hybridoma technique. Preclinical experiments showed that T cells isolated from malignant ascites support a redirected lysis of tumour cells *in vitro* by the HEA125 × CD3 trispecific antibody. Ten ovarian cancer patients were treated in a small clinical study with a 1 mg dose of antibody. Inhibition of ascites production was observed in eight out of 10 patients. A dramatic several thousand-fold increase in TNF-*α* was measured in ascites, indicating a very strong local immune stimulation. For selective recruitment of activated neutrophils and macrophages, a second construct was generated using mAb HEA125 that combines the anti-EpCAM mAb with the anti-CD64/Fc*γ*RI mAb 197. Treatment of one ovarian cancer patient with 6 × 1 mg HEA125 × 197 also reduced ascites and CA−125. Three clinical trials are in planning stage exploring dose escalations of HEA125 × CD3 and HEA125 × 197 and a combination thereof in ovarian cancer patients with malignant ascites.

U Zangemeister-Wittke (Bern, Switzerland) reported on the development of an EpCAM-specific immunotoxin called Proxinium (4D5MOC31-ETA) that currently is in a pivotal phase II/III trial for local treatment of head & neck cancer. An earlier phase I/II study has shown a tumour control rate of 88% in which 25% of injected lesions showed a complete response. Median survival of treated patients was 301 days compared with 125 days for a historic control group. The same antibody construct is now being developed for local treatment of bladder cancer (here called Vicinium®). The immunotoxin uses a stability-engineered single-chain humanised anti-EpCAM antibody fused to a subunit of the bacterial *Pseudomonas* exotoxin. The linker contains a furin cleavage site that allows for release within the endosome of the toxin after EpCAM binding and endocytosis. A conformational change of the cleaved exotoxin enables its cytosolic entry and highly efficient inhibition of the cell's protein synthesis. A novel EpCAM-directed immunotoxin is under development that has the furin cleavage site replaced by a site cleaved through matrix metalloproteinases-2 and -9, as are selectively expressed by tumour cells. This enables a dual targeting that may increase the immunotoxin's therapeutic window. *In vitro* data support that cell lines lacking MMP−9 and −2 are much less vulnerable to the immunotoxin, and that specific MMP inhibitors partially protect cells expressing the proteases from the immunotoxin. Another EpCAM-directed therapy presented by the speaker uses liposomes with single-chain anti-EpCAM antibodies linked via polyethylene glycol moieties. These long-lived liposomes are being loaded with a mix of anti-apoptotic antisense molecules specific for Bcl-2 and Bcl-XL or with doxorubicin. Xenotransplant mouse models designed for studying the targeting of [^3^H]-labelled liposomes and antitumour activity support the usefulness and potency of this approach and a combination of liposomes with anti-apoptotic and chemotherapeutic payloads.

D Herlyn (Philadelphia, USA) reviewed progress on using EpCAM as a vaccine to elicit tumour-specific T-cell and humoral immune responses. A variety of approaches were tested in small un-controlled clinical trials that use for vaccination an anti-idiotypic antibody (BR3E4), the extracellular domain of EpCAM or EpCAM encoded by an adenovirus. Evidence for specific T-cell responses and antigen spreading could be obtained.

In summary, a fair number of EpCAM-directed immunotherapies are under development as therapeutics exploiting the frequent expression of the antigen on adeno and squamous cell carcinoma (Table 1). Although three EpCAM-directed therapies are being developed for local administration because of adverse systemic effects, others are being developed for systemic use.

## THERAPEUTIC WINDOW OF THE EPCAM TARGET

Already 20 years ago, it had been realized that EpCAM is also expressed on a wide range of normal epithelia (Gottlinger *et al* 1986). Although normal cell expression is also found for other ‘tumour-associated’ antigens being used as antibody or vaccination targets, including HER-2, EGFR, CEA, MUC-1, CD20, CD52 and CD33, this circumstance has in no other case irritated researchers to the extent EpCAM's expression did. Systemic intolerability of an EpCAM-specific immunotoxin and trispecific antibodies, and acute pancreatitis, as seen with high-affinity EpCAM mAbs appears consistent with a collateral damage of normal EpCAM-expressing tissue. On the other hand, edrecolomab and adecatumumab appear well tolerated and seemingly ignore most normal EpCAM-expressing tissues. Although EpCAM is highly overexpressed in breast and ovarian cancers and expressed *de novo* in certain squamous cell carcinoma relative to their respective normal epithelia, EpCAM expression on colon carcinoma just remains at the high level of normal colonic tissue. The basis for a therapeutic window of EpCAM-directed therapies was thus lively debated at the symposium.

C Potten (Manchester, UK) set the stage for this discussion by reviewing in a very comprehensive manner the epithelial architecture, cellular dynamics, and fates of epithelial and stem cells in small intestine. P McLaughlin (Groningen, the Netherlands) investigated the consequences of targeting monoclonal antibodies to normal and tumour tissue in a transgenic murine model expressing human EpCAM. Antibodies were administered i.p. and murine tissue staining for bound antibodies performed 24–48 h later. Doses of 1, 10, 100, and 1000 *μ*g antibody were given, of which a 10 *μ*g dose would corresponded to 0.4 mgkg^−1^ in humans. This covers well the range at which acute pancreatitis was seen with mAb ING-1 in man (>1 mgkg^−1^). With increasing dose, the human EpCAM-specific IgG1 antibody USB54 stained an increasing number of epithelial tissues in transgenic mice. At the low 10 *μ*g dose, the staining of pancreas, kidney and small intestine by the antibody was most prominent. Apparently, animals showed no adverse events even at the 1-mg dose despite the apparent binding of a potentially cytotoxic human IgG1 antibody to epithelial cells of many organs. It is unclear whether the co-expressed murine EpCAM in the transgenic model could prevent cytotoxic effects of the antibody. Animal models of this kind will in the future be helpful in deciphering the basis for a differential effect of antibodies on normal *vs* tumour tissue.

The most important insights into the accessibility of EpCAM on normal epithelia *vs* tumour cells may come from recent studies identifying a number of other proteins found in close association with EpCAM within the cell membrane. F Le Naour (Villejuif, France) presented evidence on the interaction of EpCAM with tetraspanins, a family of 4-transmembrane domain proteins with diverse biological functions. One of them, CD9, was shown by crosslinking experiments to directly bind EpCAM whereas other proteins including ADAM10, integrins, G proteins, and a novel CD9 binding protein (CD9P-1) were indirectly associated. Intriguingly, loss of CD9 expression correlates with metastasis (Si and Hersey, 1993; Miyake *et al*, 1996; Zheng *et al*, 2005) and, as reported at the symposium, a strict co-localisation of CD9 and EpCAM, as seen for membranes of normal colonic epithelium, was no longer found for tumour cell membranes.

M Zoeller (Heidelberg, Germany) reported an association of EpCAM with CD44 splice variants v4–v7 and tetraspanin D6.1A. Partial cholesterol depletion destroyed these complexes, indicating their co-localisation within membrane microdomains. A novel interaction of EpCAM was found with the serine-phosphorylated version of claudin-7, a protein found in tight junctions and the basolateral side of epithelia. Crosslinking experiments implied that interaction with EpCAM was direct and does not require the EGF-like and TY domain for binding (see Figure 1). Interestingly, claudin-7 prevented EpCAM oligomerisation, which may provide a means to suppress and control EpCAM signalling in normal epithelial tissue. This is corroborated by a co-localisation of EpCAM and claudin-7 in normal tissue.

The new insights into membrane protein complexes containing EpCAM may be the key to understand both the control of EpCAM signalling in normal *vs* tumour tissue and the basis for a therapeutic window of some but not all EpCAM-directed immunotherapies. Certain antibody epitopes on EpCAM may only become accessible when accessory proteins are reduced on malignant cells or when EpCAM upon high-level overexpression outnumbers its protein partners. Subsequent oligomerisation of EpCAM may then provide a constitutive proliferation signal for tumour cells.

## CONCLUSIONS

This meeting has provided major insights into the role of EpCAM in cancer, and ongoing research promises to broaden this understanding. A focus of future research will be a more detailed understanding of EpCAM signalling in the nucleus, the regulation of EpCAM signalling as may be controlled via interaction of EpCAM with itself and other proteins in the plasma membrane, the expression and role of EpCAM on normal and cancer stem cells, and the study of clinical efficacy of a number of EpCAM-directed immunotherapies. It is hoped that this meeting review will stipulate more research on EpCAM and that a common name will be adopted by the scientific community.

## Figures and Tables

**Table 1:** EpCAM-directed immunotherapeutic approaches in clinical development

**Therapeutic (Alternative Name)**	**Class**	**Ongoing or recently completed trials**	**Dosing**	**Company**
Catumaxomab (Removab®)	Trispecific antibody; mouse IgG2a/rat IgG2b hybrid	Phase II/III in ovarian cancer Phase II in gastric cancer	i.p., escalating dose from 10–200 *μ*g	Trion Pharma/Fresenius Biotech
Proxinium® Vivendium® (VB4-845)	Immunotoxin; single-chain antibody pseudomonas exotoxin fusion	Phase II/III in head and neck cancer Phase I/II in bladder cancer	Intratumoral; up to 4 mg day^−1^ Intra-bladder	Viventia
IGN-101 (edrecolomab)	Vaccine for induction of anti-idiotypic antibody response	Phase II in various adenocarcinoma Phase II/III in non-small cell lung cancer	Multiple s.c.; 0.5 mg	Aphton
Adecatumumab (MT201)	Fully human IgG1 mAb	Phase II in metastatic breast and early-stage prostate cancer Phase I in metastatic breast, plus Taxotere	i.v.; 2 and 6 mg kg^−1^; 2-week interval i.v.; 4 and 13 mg kg^−1^ 3-week interval	Micromet, Inc./Serono
ING-1	Human engineered IgG1 mAb	Phase I in various adenocarcinoma	i.v. or s.c.; up to 6 × 1 mg kg^−1^ (MTD)	Xoma, Inc.
EMD 273 066 (huKS-IL2)	Fusion of humanized mAb KS1/4 with human IL-2	Phase I in hormone-refractory prostate cancer	i.v.; up to 6.4 mg m^2^ day^−1^ (MTD)	Lexigen, Inc./Merck KGa
Various Vaccines	Extracellular domain of EpCAM; EpCAM encoding viruses	Phase I trials in various adenocarcinoma	s.c.	Academically sponsored

EpCAM=epithelial cell adhesion activating molecule; i.p=intraperitoneal; i.v=intravenous; MTD=maximum tolerated dose; s.c=subcutaneous.

**Table 2:** Human adeno and squamous cell carcinoma so far reported to show a significant correlation of EpCAM expression with survival prognosis

Negative impact on overall survival
Node-positive breast cancer
Epithelial ovarian cancer
Gall bladder cancer
Cholangiocarcinoma
Ampullary pancreas cancer
Squamous cell cancer of esophagus
Squamous cell head & neck cancer

Positive impact on overall survival
Gastric cancer (stages I and II)
Clear cell renal cancer
Moderately differentiated stage II colon cancer
Non-small-cell lung cancer stage at stage pT2
